# Integrated analysis profiles of long non-coding RNAs reveal potential biomarkers of drug resistance in lung cancer

**DOI:** 10.18632/oncotarget.16444

**Published:** 2017-03-22

**Authors:** Wenhua Xue, Lifeng Li, Xin Tian, Zhirui Fan, Ying Yue, Chaoqi Zhang, Xianfei Ding, Xiaoqin Song, Bingjun Ma, Yunkai Zhai, Jingli Lu, Quancheng Kan, Jie Zhao

**Affiliations:** ^1^ Department of Pharmacy, The First Affiliated Hospital of Zhengzhou University, Zhengzhou 450052, Henan, China; ^2^ Biotherapy Center, The First Affiliated Hospital of Zhengzhou University, Zhengzhou 450052, Henan, China; ^3^ Department of Oncology, The First Affiliated Hospital of Zhengzhou University, Zhengzhou 450052, Henan, China; ^4^ Engineering Research Center of Digital Medicine, Zhengzhou 450052, Henan, China; ^5^ Engineering Laboratory for Digital Telemedicine Service, Zhengzhou 450052, Henan, China; ^6^ Department of General ICU, The First Affiliated Hospital of Zhengzhou University, Zhengzhou 450052, Henan, China; ^7^ Department of Clinical Laboratory, The No.7. People's Hospital in Zhengzhou, Zhengzhou 450016, Henan, China

**Keywords:** lncRNAs, lung cancer, drug resistance, bioinformatics methods

## Abstract

Lung cancer is one of the leading causes of cancer-related death. Resistance to chemotherapy and molecularly targeted therapies is a major problem that can contribute substantially to high mortality. The roles of long non-coding RNAs (lncRNAs) in drug resistance of lung cancer are insufficiently understood. Here, we identified a distinct drug resistance-related transcriptional signature and constructed a functional lncRNA-mRNA co-expression network. We found that 34 lncRNAs and 103 mRNAs have differential expression in drug resistance of lung cancer, in which 10 lncRNAs were down regulated and 24 up regulated; 49 mRNAs were down regulated and 54 up regulated. LncRNAs-mRNAs expression network analysis revealed a role for lncRNAs in modulating cancer-related pathways. We also found that two pair lncRNAs and their subnetworks were highly related to drug resistance. NR_028502.1/NR_028505.1 were found differentially co-expressed with nine mRNAs, and highly correlated with better clinical outcome. NR_030725.1/NR_030726.1 co-expressed with eleven mRNAs, and were associated with poor survival in patients with lung cancer. Our work comprehensively identified expression signature of resistance-associated lncRNAs and their inter-regulated mRNAs in lung cancer.

## INTRODUCTION

Lung cancer is a leading cause of cancer-related death worldwide [1]. Despite advances in the treatment of lung cancer, five-year survival rates remain low [2, 3]. The inevitable barrier that limits the clinical activity is the emergence of resistance, which challenges clinical long-term disease control and largely contributes to progression, recurrence and mortality of disease [4]. There are two forms of drug resistance—primary (intrinsic) resistance, which refers to lacking any treatment response to targeted therapy, and secondary (acquired) resistance, which refers to responding to treatment initially, following progression during the course of therapy [5, 6]. As the underlying mechanisms conferring the development of resistance in lung cancer remain largely unknown, the clinical examples of clearly established ways to overcome resistance are still limited [7, 8]. Therefore, it is prompting the need to develop a clear understanding of various factors that influence drug resistance in lung cancer.

Long non-coding RNAs (lncRNAs) are a class of non-protein-coding transcripts longer than 200 nucleotides [9]. Functional characterization of lncRNAs has revealed their diverse structural and regulatory roles in chromatin remodeling, imprinting, transcription, translation and epigenetic regulation [10, 11]. Thus, lncRNAs affect various human biological processes, which imply their potential as a biomarker and therapeutic target.

Several studies have recently reported the role of lncRNAs in drug resistance of lung cancer [12–15]. For example, microarray analysis revealed many lncRNAs differentially expressed in drug-resistant cell lines; lncRNA-AK126698 inhibited cisplatin-induced apoptosis in A549 cell line; lncRNA-H19 and lncRNA-BC200 mediated resistance to gefitinib; lncRNA-MEG3 increased chemosensitivity to cisplatin [13–15]. However, as these analyses only provide a limited understanding of lncRNAs, it remains a challenge to identify resistance-related lncRNAs and needs further interpret their potential biological function.

Co-expression analyses of protein-coding RNAs and lncRNAs reflect the potential function of lncRNAs. LncRNAs may regulate RNA splicing maturation, stabilize mRNAs and control nuclear/cytoplasmic shuttling of mRNAs [16]. However, most reports only analyzed the differentially expressed lncRNAs and mRNAs, no studies have reported the presence of a resistance-associated lncRNAs-mRNAs regulatory network in lung cancer.

In this study, using previously published microarray data from GEO (Gene Expression Omnibus), we identified differentially expressed transcripts (lncRNAs and mRNAs) and contructed a functional lncRNAs-mRNAs network for drug resistance in lung cancer. We found that biological processes and pathways were significantly changed by comparing tanscriptomic profiles of resistant and sensitive lung cancer lines. Clinical significance of identified lncRNAs and mRNAs was confirmed by an independent cohort of patients in GEO and TCGA (the Cancer Genome Atlas) datasets. Furthermore, we uncovered two pair lncRNAs, which were differentially expressed and could predict the survival of patients with lung cancer. This comprehensive analysis provided novel insights into lncRNAs and lncRNAs-mRNAs co-expression roles in drug resistance of lung cancer at the transcriptomic level. Selective lncRNAs might have the potential to provide valuable targets of multiple distinct processes associated with tumor resistance.

## RESULTS

### Differential expression of lncRNAs and mRNAs in drug resistance lung cancer cell lines

**Figure 1:**
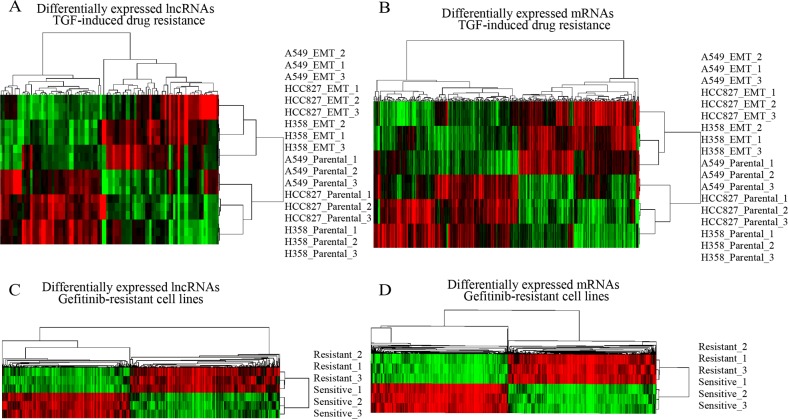
Expression differences of lncRNAs and mRNAs in drug resistance lung cancer cell lines **(A and B)** Hierarchical clustering analysis of differentially expressed lncRNAs and coding mRNAs in multidrug-resistant/sensitivity lung cancer cell lines; **(C and D)** in Gifitinib resistant lung cancer cell lines. Colors ranged from green (low expression) to red (high expression), representing the relative expression levels of lncRNAs and mRNAs.

Firstly, we analyzed the lncRNAs and mRNAs expression profiles in three different human lung cancer cell lines—A549(KRAS^G12S^-driven), HCC827 (EGFR^ΔE746-A750^-driven), and NCI-H358 (KRAS^G12C^-driven) [17]. These cells were cultured with TGFβ to acquire resistance to anticancer drugs. For this analysis, lncRNAs and mRNAs with at least 1.5 fold expression changes were selected. Under the criteria, we noted that lncRNAs transcripts were at least two times fewer than mRNAs in each cell line, suggesting that lncRNAs expression was largely conserved between multidrug-resistant cell variant and parental cell lines. We further identified a total of 101 lncRNAs and 324 mRNAs were differentially expressed (FDR<0.01; >50% increase or decrease) between multidrug-resistant cell variant and parental cell lines, consisting of 47 down regulated and 54 up regulated lncRNAs, 177 down regulated and 147 up regulated mRNAs. Unsupervised hierarchical clustering of the raw expression values of 101 lncRNAs and 324 mRNAs perfectly segregated drug resistance from parental cell lines, identifying a distinct lncRNAs and mRNAs signatures for resistance in lung cancer cells (Figure 1A, 1B).

**Figure 2:**
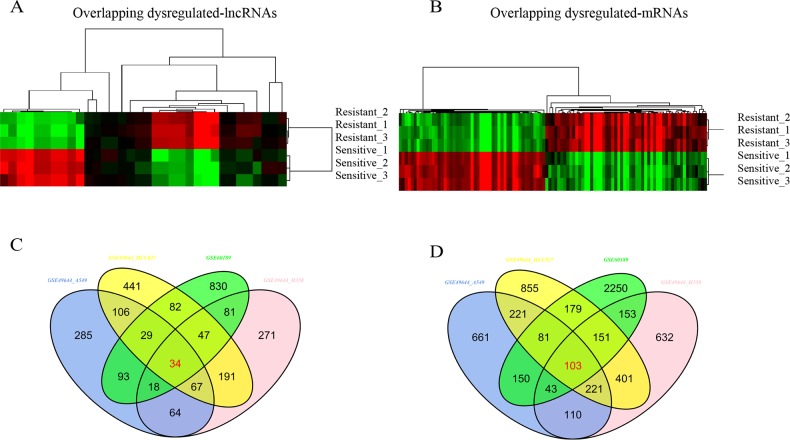
Overlapping differential expression of lncRNAs and mRNAs **(A** and **B)** Hierarchical clustering analysis of differentially expressed lncRNAs and coding mRNAs by comparison of two datasets; **(C** and **D)** Venn diagram of commonly differentially expressed genes in comparison groups. Colors ranged from green (low expression) to red (high expression), representing the relative expression levels of lncRNAs and mRNAs.

We hypothesized that if above-described lncRNAs and mRNAs are important for drug resistance, they should be regulated in other cell lines resistant to a particular drug. To test this, we profiled the lncRNAs and mRNAs expression in gefitinib-resistant and gefitinib-sensitive lung cell lines [18]. Like TGFβ-induced multidrug resistance cell lines, gefitinib-resistant cell lines had distinct transcriptional profiles compared with gefitinib-sensitive cell lines (Figure 1C, 1D). Few lncRNAs and mRNAs were overlapped with above-described genes, indicating that most differentially expressed lncRNAs and mRNAs might exclusively involve in distinct drug resistance cell lines with different regulation mechanism. By the criteria of FDR adjusted P value < 0.01 and a two-fold change, we noted 34 lncRNAs and 103 mRNAs regulated in TGF-induced resistant cell lines were also significantly changed in gefitinib-resistant cell lines. Of these differentially expressed lncRNAs, 10 were down regulated and 24 up regulated; of these mRNAs, 49 were down regulated and 54 up regulated (Figure 2, Supplementary Tables 1 and 2).

The top ranked lncRNAs in differential expression analysis, *lncRNA-ISYNA1, LINC01503, LOC641367, MBOAT1, MST1R, RCC1, TBC1D2, ZNF341* showed higher expression in resistant cell lines. The function remains largely unknown for most of the differentially expressed lncRNAs identified in our study. The top list of regulator genes is enriched for cancer-related mRNAs including *CEACAM6*,*IGFBP7*, *AGR2*, *WNT5A*, *FN1*, *IL11*. The results show that numerous lncRNAs and mRNAs are tightly associated with drug resistance. However, only a small subset of lncRNAs and mRNAs are consistently regulated and overlapped in drug resistance independent of anticancer drugs and the cell of origin.

**Figure 3:**
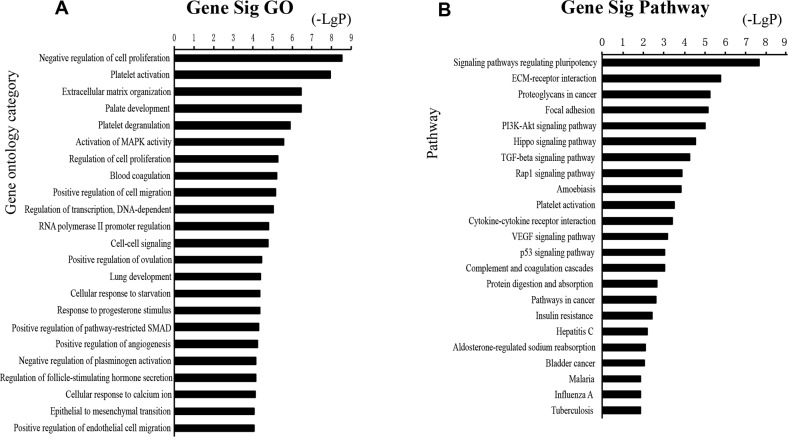
Functional analysis for the commonly differentially expressed mRNAs in drug resistance of lung cancer GO analysis and KEGG pathways of the commonly differentially expressed mRNAs.

**Figure 4:**
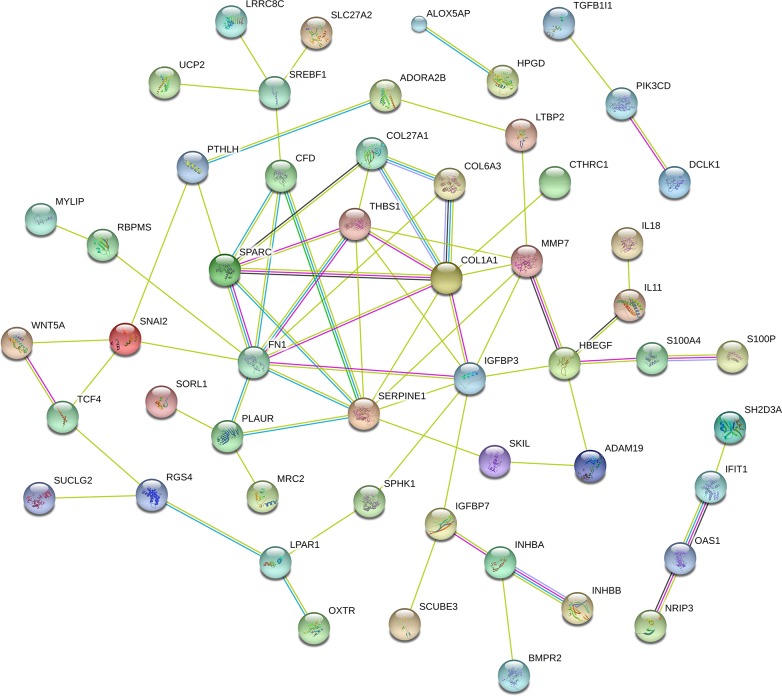
The protein-protein interaction networks constructed cytoscape software Proteins are represented with color nodes, and interactions are represented with edges.

Functional enrichment analysis based on GO Terms was performed for differentially expressed mRNAs, which significantly participated in cancer-related pathways, such as PI3K-Akt pathways, p53 pathways, VEGF pathways, Hippo pathway (Figure 3). To further mine the resistant-associated genes, we performed protein-protein interaction (PPI) network analysis. 52 genes were involved in our dataset and used to depict their complex relationship in PPI analysis (Figure 4).

### Co-expression of lncRNAs-mRNAs in drug resistance lung cancer cell lines

**Figure 5:**
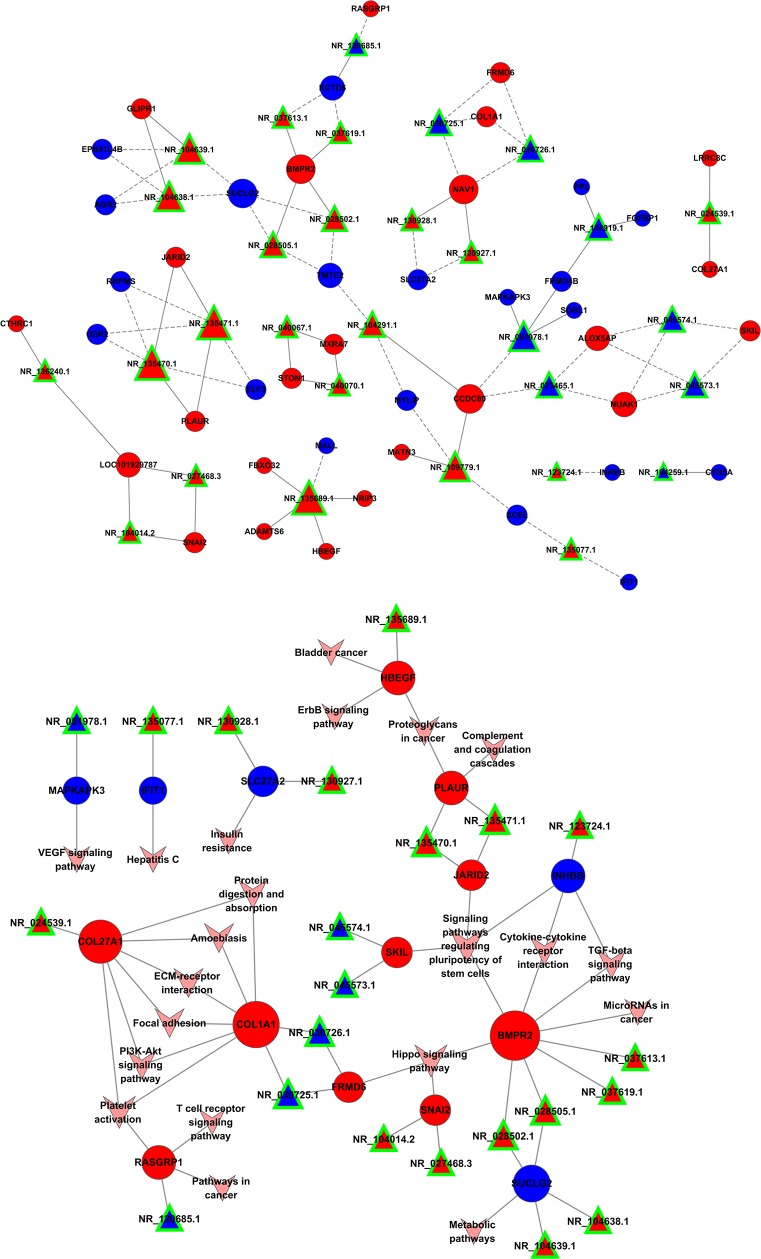
Co-expression pattern of lncRNAs-mRNAs in drug resistance of lung cancer Interaction network of lncRNAs-mRNAs. Signal pathway networks of mRNAs involved in lncRNAs-mRNAs relationships. The triangle nodes indicate lncRNAs, circular nodes indicate the mRNAs, the solid line shows their positive and direct connections, and dotted line shows a negative correlation.

A coding-non-coding gene co-expression network was used to understand roles and functional mechanisms of lncRNAs in drug resistance. Here we constructed lncRNAs-mRNAs co-expression pattern based on the correlation analysis between above-described 34 lncRNAs and 103 mRNAs across two datasets. We found that 32 lncRNAs and 81 mRNAs were inter-regulated, and most of these lncRNAs-mRNAs co-expression showed a positive correlation, suggesting that these lncRNAs-mRNAs regulatory relationships are involved in multidrug resistance of lung cancer cells (Figure 5). Additionally, one lncRNA was co-expressed with multiple mRNAs; multiple lncRNAs were also co-expressed with one mRNA, indicating a more complex regulatory relationship between lncRNAs and mRNAs in differential co-expression network. These data confirmed these lncRNAs-mRNAs regulatory relationships.

Most of the lncRNAs-associated mRNAs in the co-expression network could be assigned to functional classes relating to immune function, metabolic pathway and cancer-related pathways. We noted that these pathways signature uncovered involvement in cytokine-cytokine receptor interaction, TGFβ signaling pathway, Hippo signaling pathway, and also pointed to central roles for *COL1A1*, *COL27A1* and *BMPR2*. Though some relationship between these genes and cancer has been proposed in other systems [19], our network reveals that this mechanism may also contribute to resistance in lung cancer (Figure 5).

**Figure 6:**
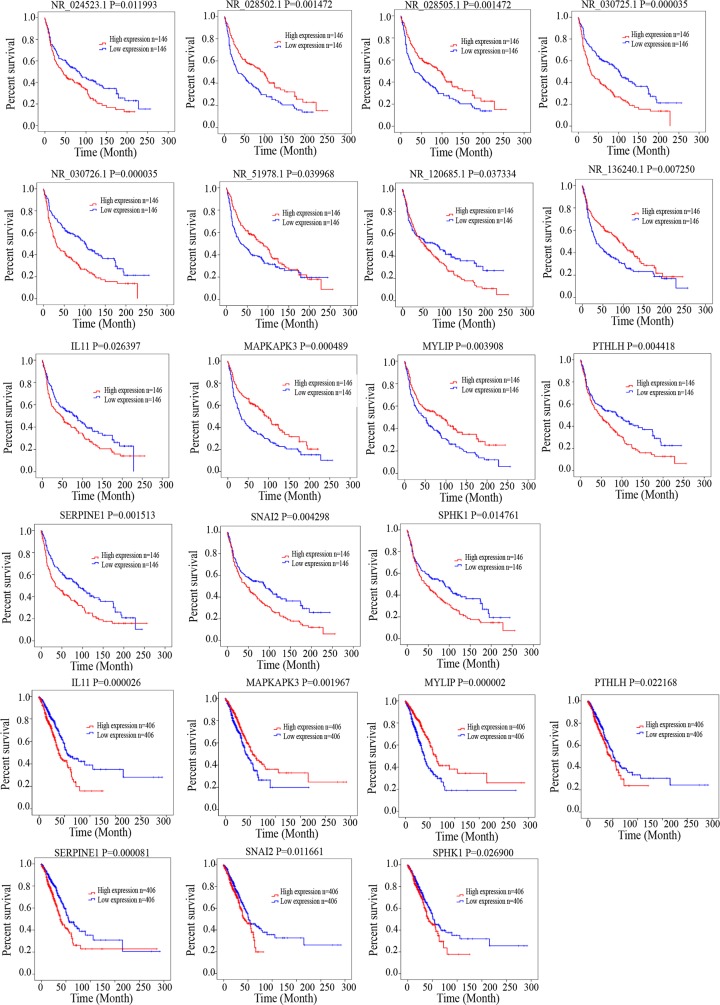
Kaplan–Meier analysis for overall survival of patients from TCGA and GEO database Log-rank test was performed to evaluate the survival differences between the two curves.

After identifying co-expressed lncRNAs-mRNAs, we examined whether these lncRNAs and mRNAs were associated with the outcome of lung cancer patients. We performed a univariate Cox regression analysis based on the expression value of these lncRNAs and mRNAs. We found that 7 mRNAs and 8 lncRNAs had significant effects on patient survival. Low levels of NR_024523.1, NR_030725.1, NR_030726.1, NR_120685.1, *IL11*,*PTHLH*, *SERPINE1*, *SNAI2*, *SPHK1* were correlated with better survival. Low levels of NR_028502.1, NR_028505.1, NR_051978.1, NR_136240.1, *MAPKAPK3*, *MYLIP* were associated with poor survival in patients with lung cancer. These results provided further evidence associating drug resistance with lncRNAs and their inter-regulated mRNAs. All statistics reflecting associations are provided in Figure 6.

### Two pairs lncRNAs involved in drug resistance lung cancer cell lines

Given that NR_028502.1, NR_028505.1, NR_030725.1, NR_030726.1 were significantly associated with the survival of lung cancer patients, we predicated their functions based on their modulated-mRNAs and network contexts. NR_028502.1 and NR_028505.1 are two transcript variants of MIR22 host gene (*MIR22HG*), which were located on 17p13.3.NR_030725.1 and NR_030726.1 are two transcript variants of regulator of chromosome condensation 1 (*RCC1*), which were located on 1p35.3. We found that lncRNA-*MIR22HG* was overrepresented in sensitive cancer cell lines, and might play a protective role in drug resistance. On the contrary, lncRNA-*RCC1* was over expressed in resistant cancer cell lines, and might play a detrimental role in drug resistance.

**Figure 7:**
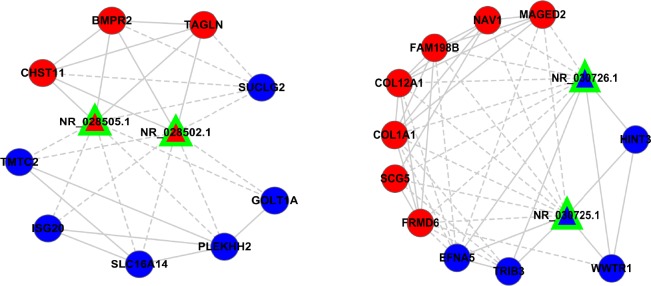
The expression of two pair lncRNAs subnetworks The expression profiles of the two pair LncRNAs and their regulatory coding genes were presented; most of the coding genes have been described in cancer.

We investigated the expression pattern of these four lncRNAs and their co-expressed mRNAs. In the sub-network, lncRNA-*MIR22HG* synergistically up regulated *CHST11*, *BMPR2*, *TAGLN*, and down regulated *SUCLG2*, *GOLT1A*, *PLEKHH2*, *SLC16A14*, *ISG20*, *TMTC2*. LncRNA-*RCC1* synergistically up regulated *HINT3*, *WWTR1*, *TRIB3*, *EFNA5*, and down regulated *FRMD6*, *SCG5*, *COL1A1*, *COL12A1*, *FAM198B*, *NAV1*, *MAGED2* in network contexts (Figure 7). We reasoned that lncRNAs affecting cancer resistance might work in part by modulating inter-regulated mRNAs in subnetwork. We found that lncRNA-*MIR22HG* and *RCC1* were presented with some genes that known to cause tumorigenesis, cancer progression and resistance in other cancer systems [20–23], suggesting that these two lncRNAs interact with genes that necessary for drug resistance in lung cancer.

## DISCUSSION

In the present study, we demonstrated a significantly altered lncRNAs and mRNAs expression profile, and contructed a functional lncRNAs-mRNAs regulatory network for drug resistance in lung cancer. We found that despite the marked alteration in lncRNAs and mRNAs expression pattern between resistant and sensitive cell lines, only 34 lncRNAs and 103 mRNAs were overlapped among different resistance profiles. Furthermore, we investigated the co-expression relationship between lncRNAs and mRNAs, and constructed lncRNAs-mRNAs co-expression pattern with inter-regulated 32 lncRNAs and 81 mRNAs. This network provided a global view of all possible resistance-associated lncRNAs and lncRNAs-regulated mRNAs in lung cancer. This observation is supported the fact, manifested by functional classification of lncRNAs-mRNAs co-expression, that dominant function of lncRNAs-regulated mRNAs is involved in cancer-related pathway, metabolic pathway and immune-related pathway in drug resistance of lung cancer.

A comprehensive database that provides functions of lncRNAs by experimental methods is still lacking. Therefore, it is essential to use bioinformatics tools and databases to predict their interactions, co-expressions, localization and function [24]. LncRNAs have been recognized as an important regulator in a wide range of biological functions. They affect their target genes to control the diversity of molecular function by targeting specific RNA or DNA sequences. In our study, the abundance of lncRNAs that are associated with drug resistance is less than their putative mRNAs, which is consistent with the notion that lncRNAs regulate gene regulatory networks in a nonlinear manner. Some lncRNAs act as a local regulator by their promoters, transcription and splicing, and some lncRNAs also can recruitment of chromatin remodeling protein complexes to influence the expression of genes [25].

Important roles for some lncRNAs are emerging in not only cellular differentiation and proliferation, but also tumor suppressive or oncogenic functions in many types of cancer [26]. Little is known about overall lncRNAs function in drug resistance, but three lung cancer-related lncRNAs (lncRNA-*AK126698*, *BC200*, *MEG3*) have been studied in detail. We note that these three lncRNAs are not included in our lncRNAs-mRNAs co-expression analysis. That is partly because we use bioinformatics approaches to identify commonly resistance-associated to both standard chemotherapeutic agents and molecularly targeted therapies, not a certain anticancer drug.

While this work emphasizes lncRNAs-specific expression, it also provides evidence those lncRNAs-relevant transcriptional networks are significantly associated with pathogenesis of cancer, a molecular feature not previous captured by differential expression analysis. Our results suggest that most functional and molecular work has focused on PI3K-Akt pathways and Hippo pathways, which were remarkably regulated by functional enrichment analysis of differentially expressed mRNA. Given this evidence, we argue that these pathways are critical in drug resistance. The conclusion also supported by previous studies that these pathways are involved in many cellular functions such as proliferation, growth, survival and metabolism by transmitting signal transduction events in cancer [27, 28]. Our results also showed that these pathways were inter-linked in the pathway-network analysis, which provided a cellular and molecular network of drug resistance, and were also supported by data from experimental results [29–31]. Beyond implication for mRNA-associated pathway, the importance of three mRNAs, *COL1A1*, *COL27A1* and *BMPR2*, were captured in our network analysis. These three mRNAs were highly expressed in drug resistance cell lines. However, as the role of these three mRNAs has not been reported to be involved in the pathogenesis of tumor, the exact mechanism of these genes sharing the role in drug resistance still requires in-depth research. Our integrative networks, accompanied by these functional data, support the hypothesis that differentially expressed lncRNAs and their inter-regulated mRNAs are an important contributor to drug resistance in lung cancer.

Furthermore, we highlighted lncRNA-*MIR22HG* and *RCC1* are two critical lncRNAs that were co-expressed with multiple mRNAs in drug resistance. Previous work has demonstrated lncRNA-*MIR22HG* is a surrogate indicator of specific cell stress that has been clearly implicated in different phases of tumorigenesis [32, 33]. Their biological mechanism has not been elucidated and validated by experimental studies; it is still not clear how these lncRNAs function in drug resistance of lung cancer. However, publicly available microarray datasets revealed that high levels of lncRNA-*MIR22HG* expression correlated with better survival for patients with lung cancer; lncRNA-*RCC1* was associated with poor clinical outcome. These results suggest that lncRNA-*MIR22HG* and *RCC1* and their target gene, could contribute to the mechanisms underlying the drug resistance of lung cancer.

Notably, these two lncRNAs-regulated mRNAs in our analysis were also documented; *MIR22HG* up regulated three and down regulated six differentially expressed mRNAs, and *RCC1* up regulated four and down regulated seven differentially expressed target genes. Previous work has demonstrated lncRNAs perform a *cis* or *trans* regulatory role on its target genes [34]. Although regulated protein-coding genes have not been revealed in drug resistance, some of them have been described in other tumors. For example, *GOLT1A* knockdown restored tamoxifen sensitivity in TamR cell; low *GOLT1A* expression was associated with good prognosis for breast cancer [35]. *CHST11* might play a direct role in progression of breast cancer and that its expression was controlled by DNA methylation [36]. *BMPR-II* was observed in the peripheral blood of breast cancer patients especially in the advanced-stage of the disease [20]. *TAGLN* was upregulated in osteosarcoma cell lines and tumor tissue [21]. Melanoma antigen D2 (*MAGED*2) was recognized as a cancer diagnostic marker by changing intracellular localization and shuttling during cell cycle progression and in response to cellular stress [22]. Genetic inhibition of *TRIB3* enhanced tumorigenesis by regulating Akt pathway [22]. *WWTR1*, downstream effectors of Hippo pathways, was associated with worse prognostic outcome in various cancers [23]. These findings emphasize the importance of lncRNA-*MIR22HG* and *RCC1*, and necessity of studying system-level gene interactions in order to understand and uncover the complex mechanisms associated with drug resistance in lung cancer.

Although we identified differential lncRNAs-mRNAs expression pattern, the detailed mechanism underlying drug resistance remains unclear. The fact that lncRNAs impact gene regulation by lncRNAs-mRNAs interaction doesn't mean lncRNAs does not have other regulatory mechanisms. Our analysis do not rule out the possibility the lncRNAs dissected in this study have other functions such as chromatin remodeling, promoter demethylation, microRNA silencing. Future studies should use anti-lncRNAs knockdown to identify protein-coding loci and assess functional relationship between lncRNAs and co-expressed mRNAs. Therefore, we believe a comprehensive understanding of drug resistance requires further investigation of the mechanisms underlying its transcriptional networks. In conclusion, our study revealed two lncRNAs and lncRNAs-mRNAs co-expression pattern are associated with drug resistance in lung cancer, which might potentially be used as an independent marker to predict resistance.

## MATERIALS AND METHODS

### Microarray data of mRNAs and lncRNAs in drug resistance of lung cancer

The lncRNAs and mRNAs expression dataset was retrieved and download from the NCBI Gene Expression Omnibus (GEO) database by searching drug resistance in lung cancer. After screening, we chose two datasets with series accession number GSE49644 and GSE60189 for our analysis (http://www.ncbi.nlm.nih.gov/geo/query/acc.cgi?acc=GSE49644, http://www.ncbi.nlm.nih.gov/geo/query/acc.cgi?acc=GSE60189). These datasets consist of gefitinib resistance in PC9/gef cell lines [18], and TGFβ-induced multidrug resistance in A549, HCC827 and NCI-H358 cell lines [17]. Samples in two datasets were primarily analyzed with Affymetrix Human Genome U133 Plus 2.0 Array.

The raw data files of GSE49644 and GSE60189 were performed with Affymetrix Expression Console. Probe signal values were converted to log2 values, and analyzed according to array annotation files (Supplementary Table 3). In addition, to profile the lncRNA expression in the present data, the genes from downloaded datasets were compared with NCBI RefSeq transcript using BLAST program. Finally, lncRNA candidates were obtained and used for further analysis. We renamed these lncRNA based on NCBI database.

### Identification of resistance-associated mRNAs and lncRNAs

We performed differential expression analysis by comparing mRNAs and lncRNAs expression in three different lung cancer cell lines, which acquire multidrug resistance by cultured with TGFβ. The false discovery rate (FDR) < 0.01 and >50% increase or decrease were used as the cut-off for significantly differentially expressed genes. We then compared mRNAs and lncRNAs profiles in gefitinib-resistant and gefitinib-sensitive lung cancer cells. After overlapping these gene profiles, 34 lncRNAs and 103 mRNAs were found to be commonly differentially expressed in drug resistance of lung cancer. Differentially expressed genes then subjected to gene ontology (GO) and enrichment analyses to determine significantly regulated functions. Kyoto Encyclopedia of Genes and Genomes (KEGG) pathway analysis was used to locate the significant enrichment pathway. The annotation of protein cellular localization and biological function was performed by using protein-protein interaction (PPI) network.

### Co-expression analysis

Most of the lncRNAs have not yet been functionally annotated; focus has been on understanding how lncRNAs are regulated to influence protein-coding genes. Thus, functional annotations of their co-expressed mRNAs are used to predict functions of lncRNAs. By estimating across array, we generated lncRNAs-mRNAs co-expression network based on the expression value between differentially expressed lncRNAs and mRNAs. Significant correlation pairs were used to construct the network based on Pearson correlation coefficients and corresponding FDR. The connection matrix A was constructed. Factor analysis is used to find Characteristic-Value by calculating the distance between the genes. Characteristic-Value is the contributions of the gene i to lncRNA j. This model was defined as *λx_i_* = *a*_1i _x_1_ + *a*_2i_x_2_ +…+ *a_ni_x_n_*, where x is eigenvector centrality. This model can be converted to *A^t^* · *x* = *λx*, where A is an n×n matrix. This equation has n observations on n variables. Then rank the Characteristic-Value, maximum value is the core mRNA or lncRNA. The results M (i, j) define the edges of the graph and an edge connecting the nodes. In the graphic, triangle nodes as lncRNAs, circular nodes as the mRNAs, the solid line as positive and direct connections, and dotted line as a negative correlation. We identified degree of gene centrality using the number of links from one node to another, which depicts its relative importance in biological function.

### Survival analysis

We further investigated whether the co-expressed lncRNAs-mRNAs was associated with the potential good or poor outcome. Thus, we first collected lung cancer samples with lncRNAs and mRNAs expression and clinical information from TCGA and GEO database (GSE30219) [37]. In total, 812 samples in TCGA and 292 samples in GEO database with clinical follow-up information were obtained for further analysis. To analyze the correlation between gene expression signatures and the corresponding overall survival in lung cancer, only the data with survival information were used. The gene expression was labeled as high or low using dichotomy method. To determine which gene could potentially be of functional significance in lung cancer prognosis, univariate Cox regression analysis was performed to examine the relationship between the gene expression levels in patients and the overall survival. Differences in the overall survival between the two groups were estimated and compared by the Kaplan–Meier method with log-rank test.

### Statistical analyses

The differentially expressed of lncRNAs and mRNAs between sensitive and resistant lung cancer cell lines were compared using the Bioconductor package limma (version3.26.1) and R (version 3.2.2) software. The FDR was also calculated to correct the P value for multiple testing. P values less than 0.05 were considered statistically significant.

## SUPPLEMENTARY MATERIALS TABLES








