# Supplementary material for: Integrated analysis profiles of long non-coding RNAs reveal potential biomarkers of drug resistance in lung cancer
Source: Oncotarget. 2017 Mar 22;8(38):62868–79. doi: 10.18632/oncotarget.16444 (PMC5609887; doi:10.18632/oncotarget.16444)
Supplement: Supplementary file 1 [file oncotarget-08-62868-s001.pdf]

Integrated analysis profiles of long non-coding RNAs reveal potential biomarkers of drug resistance in lung cancer

SUPPLEMENTARY TABLES

Supplementary Table 1: Overlapping differential expression of lncRNAs in drug resistance of lung cancer. (XLS).
See Supplementary File 1

Supplementary Table 2: Overlapping differential expression of mRNAs in drug resistance of lung cancer. (XLS).
See Supplementary File 2

Supplementary Table 3: U133_refseq_NR lncRNA. (XLS).
See Supplementary File 3
